# Cannabis-based medicines and the perioperative physician

**DOI:** 10.1186/s13741-019-0127-x

**Published:** 2019-12-06

**Authors:** Patrick Tapley, Suzanne Kellett

**Affiliations:** 1 Department of Anaesthesia, Sunnybrook Health Sciences Centre, Bayview Avenue, Toronto, Canada; 2 Shackleton Department of Anaesthesia, University Hospital Southampton, Tremona Road, Southampton, UK

**Keywords:** Pharmacology, Cannabis, Cannabinoids, Medical marijuana

## Abstract

The increasing availability of cannabis for both recreational and medicinal purposes means that anaesthetists will encounter an increasing number of patients taking cannabis-based medications. The existing evidence base is conflicted and incomplete regarding the indications, interactions and long-term effects of these substances. Globally, most doctors have had little education regarding the pharmacology of cannabis-based medicines, despite the endocannabinoid system being one of the most widespread in the human body. Much is unknown, and much is to be decided, including clarifying definitions and nomenclature, and therapeutic indications and dosing. Anaesthetists, Intensivists, Pain and Perioperative physicians will want to contribute to this evidence base and attempt to harness such therapeutic benefits in terms of pain relief and opiate-avoidance, anti-emesis and seizure control. We present a summary of the pharmacology of cannabis-based medicines including anaesthetic interactions and implications, to assist colleagues encountering these medicines in clinical practice.

## Introduction

Cannabis use for medicinal purposes was first documented in 2900 BC in China, when Emperor Shen Nong described benefit for rheumatism and malaria (Pertwee 2015) and later in Ancient Egyptian texts (Pertwee 2015; Zlas et al. 1993). In the United Kingdom (UK), Queen Victoria’s personal physician Sir John Russell Reynolds issued a tincture containing cannabis for her Majesty’s menstrual cramps (David 2017), subsequently publishing his 30 years’ worth of experience with the drug (Reynolds 1890).

Discussion in medical journals, the mainstream and social media around the use of cannabis for medicinal and non-medicinal purposes has increased recently, especially following the legalisation of cannabis for recreational use in Canada (Government of Canada 2018a) and the UK government’s decision to make cannabis-based medicines (CBMs) available for prescription by doctors on the specialist register (Department of Health and Social Care 2018).

The actual, social and economic legitimisation of cannabis and its medicinal derivatives makes it likely increasing numbers of patients will present on this class of medicines. Perioperative physicians will require a sound understanding of their pharmacology and evidence base, and may wish to exploit this group of compounds for therapeutic purposes in the perioperative period.

A search of Pubmed was conducted in February 2019 utilising the search terms cannab* and the AND function for the following search terms individually; anaes*, marijuana, pain, nausea, surgery and pharmaco*. Abstracts were then screened for their applicability, with full texts reviewed. This was supplemented by a review of recent publications from governmental and regulatory organisations relating to CBMs, with backward reference searching. A search of individual governmental websites looking for legislation around cannabis and cannabinoid use was also undertaken in September 2019.

The pharmacology of novel psychoactive compounds (“legal highs”) is outside the scope of this review.

## The global position

The availability of CBMs varies geographically and there is no global consensus on how cannabis and CBMs should be regulated. International stakeholders and regulators, including the United Nations (International Drug Policy Consortium 2016; Transnational Institute and Global Drug Policy Observatory 2016) and World Health Organisation (WHO) have been inconsistent in their approach. The WHO’s Expert Committee on Drug Dependences’ recent review (Ghebreyesus 2019) recommended to the United Nations Office on Drugs and Crime (UNODC) that the rescheduling within the International Drug Control Conventions occurs for cannabis and cannabis resin, dronabinol, tetrahydrocannabinol and extracts and tinctures of cannabis. They also repeated their recommendation to remove cannabidiol (CBD) preparations (with not more than 0.2% delta-9-THC) from the International Drug Control Conventions. The UNODC subsequently delayed its vote on these recommendations, but despite this, many countries are proceeding to legalise or reschedule cannabis and/or CBMs, broadening public availability, with the UK the most recent country to reschedule CBMs. Table 1 details the current status of cannabis and CBMs in selected countries for medical use. The recreational use of cannabis is currently legalised in Uruguay, Canada and certain states within the USA (United Nations Office on Drugs and Crime 2019).

**Table 1:** Current legal status of Cannabis and CBMs in selected countries

Continent	Country	Year approved	Notes
North America	America (United Nations Office on Drugs and Crime 2019; Mead 2017; US Food and Drug Administration 2016; Aguilar et al. 2018)	1996-2017	Cannabis illegal under federal law, individual states have legalised to varying degrees Medical use: -Legalised by 33 states -FDA has approved dronabinol (Marinol ®, Syndros® ), nabilone (Cesamet ®,) and cannabidiol (Epidiolex ®) (CBMs) for specific indications, but cannabis plant not FDA approved Recreational use: -Legalised by 10 states (Alaska, California, Colorado, Maine, Massachusetts, Michigan, Nevada, Oregon, Vermont, Washington) and District of Colombia for recreational use -Illinois to be legalised January 1 2020 -Multiple states have bills in session to legalise recreational and medicinal cannabis and its products -“Decriminalised” in numerous states (ie avoid jail term)
	Canada (United Nations Office on Drugs and Crime 2019; Aguilar et al. 2018; Government of Canada 2018b; Health Canada 2018)	1999/2018	Medical use: -Regulated by federal government -Healthcare practitioner authorisation required -Patients can access cannabis through federally licensed seller, designate someone to produce, or grow their own. Recreational use: -Cannabis for recreation legalised October 2018 under Cannabis Act -Cannabis act permits sale of cannabis oil, fresh cannabis, dried cannabis, cannabis plants and seeds -Retailers must be authorised to sell cannabis
	Mexico (Aguilar et al. 2018; Government of Mexico 2018a; Government of Mexico 2018b; Government of Mexico 2018c)	2017	Medical use: Cannabis use allowed for medical and scientific purposes Application required for import and use of cannabis and CBMs for medical purposes Products with < 1% THC recently marketed under Government authorisation with no prescription required
South America	Uruguay (United Nations Office on Drugs and Crime 2019; Aguilar et al. 2018; Uruguay Government 2018)	2013	Medical use: -Prescription required for CBM for medicinal use -Medicinal cannabis not authorised to be sold -CMs accessible via specialist prescription Recreational Use: -Cannabis legalised and market regulated from December 2013 with strict controls
	Chile (Aguilar et al. 2018)	2015	Medical use: -Medical prescription required for medicinal cannabis -CBM can be imported under special license
	Brazil (Aguilar et al. 2018; Brazilian Government 2016)	2014	Medical use: -Imports of medications based on CBD oil for medical and therapeutic use for patients with prescription allowed -Nabiximols (Sativex ®) licensed for MS -Cannabis plant prescription prohibited
	Argentina (Aguilar et al. 2018; Argentinian government 2018; Government of Argentina 2019)	2017	Medical use: -Prescription and government authorisation required through ministry of health -State produced pharmaceuticals for domestic market -Cannabis oils legal for import
Oceania	Australia (Aguilar et al. 2018; Australian Government Department of Health 2018; Therapeutic Goods Administration 2017)	2016	Medical use: -cultivation, manufacture, prescribing and dispensing of medicinal cannabis products for patients now legal -Access via authorised prescriber program, or special access scheme on individual patient basis. Must be prescribed -Cannabis, THC, nabiximols (Sativex ®), dronabinol (Marinol ®), nabilone (Cesamet ®, Canemes ®) listed under schedule 8, require prescription -Cannabidiol listed under schedule 4, requires prescription
	New Zealand (Aguilar et al. 2018; New Zealand Parliament 2018; New Zealand Ministry of Health 2018)	1977	Medical use: -Prescription required for most cannabis-based products, some require ministerial approval -Nabiximols (Sativex ®)and CBD based products available without ministerial approval -Bill currently passing through NZ parliament to improve access
Europe	Netherlands (Aguilar et al. 2018; Office of Medicinal Cannabis 2018; Government of the Netherlands 2019; Office of Medicinal Cannabis Government of the Netherlands 2019)	2000	Medical use: -Medicinal use legalised in 2000 -Office of Medicinal Cannabis government agency responsible for supplying medicinal cannabis to pharmacies, prescription required -5 compositions produced, with varying strengths of THC and CBD Recreational use: -Illegal, but smoking cannabis under strict conditions allowed
	Germany (Aguilar et al. 2018; Broich 2018)	2017	Medical use: -Medicinal cannabis products able to be prescribed by physician -CBMs included in range of medications covered by public and private health insurance
	Poland (Aguilar et al. 2018)	2017	Medical use: -Medicinal cannabis sold in registered pharmacies. -Patients need permission from pharmaceutical inspector and physician
	France (Aguilar et al. 2018; National Agency for the Safety of Medicines and Health Products 2013)	2013	Medical use: -Law changed to allow marketing authorisation of products containing cannabis or its derivatives -Dronabinol (Marinol ®) approved and marketed -Nabiximols (Sativex ®) approved but not marketed due to price disagreements
	Ireland (Aguilar et al. 2018; Department of Health Ireland 2018; Health Products Regualtory Authority 2017)		Medical use: -Cannabis for medical use access programme enables prescribing of Cannabis for medical use by Medical Consultant -Authorised CBMs (nabiximols (Sativex ®) , dronabinol (Marinol ®), nabilone (Cesamet ®, Canemes ®) should be used in first instance with prescription -Cannabis (plant or extracts not authorised as medicine) considered as treatment option -Cannabidiol (Epidiolex ®) not currently authorised
	UK (National Institute for Health and Care Excellence 2019; Dame Sally Davies 2018; National Health Service 2019)	2018	Medical use: -CBMs recently rescheduled, only available via prescription from doctor on the specialist register **-**Nabilone and Nabiximols (Sativex ®) currently licensed but the latter is not approved by NICE -Dronabinol not available as a licensed medicine -Cannabidiol not classed as CBM, and not controlled. Epidiolex® currently in licensing process. -NICE draft guidance for prescription of CBMs currently open for public consultation
Middle East	Israel (Aguilar et al. 2018)	1992	Medical use: -Medical Cannabis Unit established for the regulation of Cannabis -Specialist physicians apply for Medical Cannabis permit on behalf of patient - > 40,000 patients receiving medicinal cannabis
Asia	Japan (Aguilar et al. 2018)		Prohibited
	Pakistan (Aguilar et al. 2018)		Prohibited
	Philippines (Aguilar et al. 2018; Republic of Phillipines House of Representatives 2018)	2016	Medical use: -Cannabis allowed with prior authorisation from a doctor and treatment delivered in dedicated centres -Covers all forms of Cannabis, no specific mention CBMs
	India (Aguilar et al. 2018; Indian Council of Medical Research 2018)		Medical use: -Legal provisions for medicinal use yet to be implemented -Council of Scientific and industrial research currently undertaking research into medical benefits
Africa	South Africa (Department of Agriculture, Forestry and Fisheries 2018; Constitutional Court of South Africa 2017; Medicines Control Council 2016)	2018	Medical use: -Special authorisation from Medical Control Council by physician can be requested for prescription of medicinal cannabis products -Cannabidiol preparations excluded from Medicines and related substances act

## Definitions of cannabis and cannabinoids

### Cannabis

The *Cannabis* genus encompasses three major species; *Cannabis sativa*, *Cannabis indica* and *Cannabis ruderalis*. The number of identifiable extractable compounds has increased dramatically from 60 (Ashton 1999) to over 500 in the last 20 years (Beaulieu et al. 2016), of which over 100 are cannabinoids (Bie et al. 2018).

### Cannabinoids

Cannabinoids are endogenous in humans, animals and plants, or synthetically produced, acting as ligands at the cannabinoid receptors. Cannabinoids can be psychoactive, for example delta-9-tetrahydrocannabinol (d9THC), delta-8-tetrahydrocannabinol (d8THC), cannabinol (CBN) or non-psychoactive, for example cannabidiol (CBD). Table 2 lists their classification, as well as examples of currently available CBMs (Pertwee 2015; Beaulieu et al. 2016; Zendulka et al. 2016; Yeon Kong et al. 2018; Hauser et al. 2018a; Barnes 2018; National Institute for Health and Care Excellence 2014; Rice and Cameron 2017; Krcevski-Skvarc et al. 2018).

**Table 2:** Classification of cannabinoids and some commercially available preparation

Group	Where found	Major known Compounds	Substance found in	Trade name	Route of administration	Current uses
Endo -cannibinoid	Endogenous within body	-Arachidonoyl ethanolamide (Anandamide) -2-arachidonylglycerol (2-AG) -Docosatetraenoyl-ethanolamide (DEA), -N-arachidonyldopamine (NADA), -Virodhamine, -2-arachidonylglyceryl, -noladin ether -dihomo- gamma-linolenoylethanolamide (HEA).				
Synthetic cannabinoids	Chemically synthesised	Synthetic THC -(-) trans isomer of delta 9 THC	Dronabinol	Marinol®	Oral capsule (formulated in sesame oil)	US; FDA approved; -anorexia associated weight loss in AIDS patients -chemo induced nausea and vomiting * UK; not available
				Syndros®	Liquid formulation	US; FDA approved - anorexia associated weight loss in AIDS patients -chemo induced nausea and vomiting *
		Synthetic THC analogue (single molecule)	Nabilone	Cesamet®	Oral capsule	US; FDA approved chemo induced nausea and vomiting UK; -chemo induced nausea and vomiting *
		Variety; mainly aminoalkylindole derivatives ie JWH-018, UR-144, PB-22	Legal highs	Spice, K2, eclipse	Oral Smoked	
Phyto -cannibinoid (plant derived)	Cannabis genus; -cannabis sativa, -cannbis indica -cannabis ruderalis	THC content varies 1-22% CBD content varies 0.05-9%	Cannabis; Marijuana		Inhalation (commonly smoked)	UK; Illegal US; legalised for medical use in 29 states, recreational use in 9 states
		THC content varies 1-22% CBD content varies 0.05-9%	Cananbis; Hashish		Oral	UK; Illegal US; legalised for medical use in 29 states, recreational use in 9 states
		Pure CBD (no psychoactive properties)	Cannabidiol		Oral capsule Oromucosal spray	
				Epidiolex	Oil	US;FDA approved for treatment resistant epilepsy
		2.7mg THC and 2.5mg CBD per spray	Nabiximols	Sativex®	Oromucosal spray	US; not approved. Approved in 30 other coutnrie UK; MHRA but not NICE approved. MS induced spasticity
		2.5mg THC, 1.25mg CBD	Cannador		Oral capsule	German based research only

*not responded to conventional antiemetics

## Pharmacology of cannabinoids

### Mode of action

The endocannabinoid system consists of both cannabinoid (CB) receptors and neurotransmitters, the plasma concentrations of which are normally at low levels. They are synthesised in the postsynaptic neurone (Hosking and Zajicek 2008) in response to stimuli including pain, stress, inflammation and are involved in the homeostasis of various body systems (Pertwee 2015). Antinociceptive effects occur via their actions as retrograde transmitters at presynaptic inhibitory CB_1_ receptors (Hauser et al. 2018a). Both CB_1_ and CB_2_ receptors are G protein coupled receptors (G_i_,G_o_) with stimulation reducing cAMP production through the inhibition of adenylyl cyclase, resulting in an action on voltage gated calcium and potassium channels depressing neuronal excitability and reducing neurotransmitter release (Zendulka et al. 2016; Hauser et al. 2018a; Hosking and Zajicek 2008).

CB_1_ receptors are found in the cortex (thalamus, medulla, periaqueductal gray matter, descending pain pathways), spinal cord (descending pain pathways, dorsal horn) and peripherally on primary afferent sensory neurones where they outnumber the mu receptor, suggesting a potential mechanism for the modulation and treatment of neuropathic pain (Kumar et al. 2001).

CB_2_ receptors are involved in immunomodulation, with receptors distributed in the spleen, macrophages and Kupffer cells. It is increasingly recognised that the endocannabinoid system plays a crucial role in the maintenance of microglial activity through actions at CB1 and CB2 receptors, reducing neuro-inflammation (Bie et al. 2018; Bilkei-Gorzo et al. 2018). Relatively few CB_2_ receptors are found in the nervous system (Lucas et al. 2018), but they are inducible in the dorsal horn following inflammation or injury, with increased receptor concentration found in neuropathic pain models and receptor activation limiting the acute inflammatory process contributing to nociceptor sensitisation (Bie et al. 2018; Hosking and Zajicek 2008).

Exogenous and endogenous cannabinoids have differing effects at the CB_1_ and CB_2_ receptors. THC is an agonist at both, whilst CBD is a non-competitive antagonist at CB_1_ receptors at high concentrations, an inverse agonist at CB_2_ receptors and causes allosteric modulation of both CB receptors (Pertwee 2015; Lucas et al. 2018; Expert Committee on Drug Dependence 2018). The cannabinoid compounds, particularly CBD, have additional actions within the nervous system through signalling at a multitude of other receptors. This includes adenosine, serotonergic, adrenergic, nicotinic acetylcholine, glycine, nuclear peroxisome proliferator activated receptors (PPARs) and transient receptor potential (TPRV) ion channels (Capsaicin target). Anaesthetists should also note their actions at the opioid, NMDA and gamma amino butyric acid (GABA) receptors (Zendulka et al. 2016; Hauser et al. 2018a; Expert Committee on Drug Dependence 2018; Meng et al. 2017; Koppel et al. 2014).

### Opioid system interaction

The cannabinoid and opioid systems are closely linked, with the activation of both opioid and cannabinoid receptors mediating common intracellular signalling mechanisms (Manzanares et al. 1999; Abrams et al. 2011; Scavone et al. 2013; Cohen et al. 2019; Pertwee et al. 2010). Opioid and cannabinoid receptors are found within the same cells and neurones in the central nervous system, with cannabinoids acting at kappa and delta receptors to increase endogenous opioid synthesis and release. Notably, the administration of opioid antagonists has been shown to block some of the effects of delta 9THC (Manzanares et al. 1999). The presence of opioid and cannabinoid receptors in noradrenergic pathways may have a role in the treatment of opiate withdrawal (Scavone et al. 2013).

### NMDA system interaction

The NMDA receptor NR1 subunit is closely coupled to CB1 receptors, with the histidine triad nucleotide binding protein 1 (HINT 1) thought to be the pivotal modulator, exerting a negative control on NMDA receptors. HINT-1 gene deletion results in loss of CB1 inhibition of the NMDA receptor (Rodríguez-Muñoz et al. 2016). CB1 receptors have both presynaptic (reduced release of glutamate into synaptic cleft) and post-synaptic (intracellular NMDA signalling) effects (Rodríguez-Muñoz et al. 2016).

NMDA receptor activity stimulates the release of endocannabinoids, resulting in negative feedback reducing NMDA receptor numbers. It has been postulated that exo-cannabinoids are more potent inhibitors of the NMDA receptor than endocannabinoids (Pacheco et al. 2019; Ferreira et al. 2018), with exo-cannabinoids causing neural dysfunction and NMDA receptor hypofunction through alteration in the balance of NMDA-CB receptor regulation (Rodríguez-Muñoz et al. 2016).

The endocannabinoid system also regulates NMDA receptor activity by preventing over activation, neuroprotection from excitotoxicity and downregulating their activity (Rodríguez-Muñoz et al. 2016; Pacheco et al. 2019; Sánchez-Blázquez et al. 2014).

### Gamma amino butyric acid

Gamma amino butyric acid (GABA) and CB1 receptors are closely localised in multiple cortical regions, including the hypothalamus, hippocampus and cortex (Cohen et al. 2019; Lotsch et al. 2018). CB1 receptors are expressed on GABAergic neurons, helping to regulate astrocyte and microglial activity, and hence neuroinflammation (Bilkei-Gorzo et al. 2018).

In preclinical studies, cannabinoids inhibit GABA release, through activation of CB1 receptors (Pertwee 2015; Laaris et al. 2011). They inhibit GABA uptake from the CNS extracellular space (Laaris et al. 2011), and cause allosteric modulation of GABA receptors (Bakas et al. 2017). Limited human studies show altered levels of GABAergic functions with chronic cannabis use, which may contribute to psychological effects (Cohen et al. 2019).

## Pharmacokinetics

### Absorption

The absorption of vaporised cannabinoids is rapid, with peak plasma concentrations observed within 10 min. THC’s bioavailability after inhalation ranges from 10 to 35%, and CBD 31% varying with device used and size of the particles (Kumar et al. 2001; Lucas et al. 2018; Karschner et al. 2011).

Oral bioavailability of CBM is low, at 2–20% for both CBD and THC (Lucas et al. 2018; Karschner et al. 2011; Anderson and Chan 2016) mainly due to extensive first pass metabolism (Lucas et al. 2018). Onset of action is 0.5–2 h due to slow intestinal absorption resulting in a longer duration of action (Kumar et al. 2001; Bridgeman and Abazia 2017).

An oromucosal spray preparation (nabiximols; Sativex ®) has a reported bioavailability similar to oral THC or intermediate between the oral and inhaled routes (Lucas et al. 2018; Expert Committee on Drug Dependence 2018; Karschner et al. 2011; Anderson and Chan 2016).

Transdermal administration is reported, with the permeability of CBD and CBN higher than d9THC and d8THC (Therapeutic Goods Administration 2017), but their hydrophobic nature means transdermal absorption is poor and requires permeation enhancement (Lucas et al. 2018).

### Distribution

Volume of distribution varies by cannabinoid studied, with a V_D_ of 32 L/kg for CBD (intravenous administration), and 3.4 L/kg for THC (inhalation administration) (Lucas et al. 2018), which is said to follow a three-compartment model (Heuberger et al. 2015). Cannabinoids are highly lipid soluble (Kumar et al. 2001) with rapid penetration through the blood–brain barrier (Ashton 1999), the placenta and into breast milk (Lucas et al. 2018). This also leads to accumulation in fatty tissue, with continued activity following cessation.

### Metabolism

The cannabinoids are mainly hydroxylated and glucuronidated in the liver by the cytochrome P450 family of isoenzymes (Kumar et al. 2001; Lucas et al. 2018; Karschner et al. 2011; Ujváry and Hanuš 2016). Some metabolites are equipotent to the parental compounds (Yeon Kong et al. 2018; Rong et al. 2017). THC is metabolised to over 80 metabolites by various isoenzymes, including CYP1A1, CYP1A2, CYP1B1, CYP2B6, CYP2A6, CYP2C9 and CYP3A4. Inhibition of CYP3A4 may result in clinically apparent interactions with oxycodone (Pertwee 2015; Zendulka et al. 2016; Hauser et al. 2018a; Lucas et al. 2018).

CBD is metabolised to over 100 metabolites by isoenzymes CYP1A1, CYP1A2, CYP3A4, CYP2C9 and CYP2D6, the most abundant metabolite being the hydroxylated 7-COOH CBD derivative (Lucas et al. 2018; Ujváry and Hanuš 2016). Inhibition of CYP2D6 and CYP3A4 results in interactions with oxycodone, benzodiazepines and haloperidol (Hauser et al. 2018a; Karschner et al. 2011; Ujváry and Hanuš 2016). Oral CBD increases clobazam (and active metabolite) plasma levels (CYP2C19 interaction) (Ujváry and Hanuš 2016), resulting in dose reductions of clobazam in recent randomised controlled trial (RCTs) (Thiele et al. 2018). Prolonged use of CBD results in CYP1A1 induction (Ujváry and Hanuš 2016).

Cannabinol is metabolised via CYP2C9 and CYP3A4, with no evidence cytochrome P450 interactions (Zendulka et al. 2016).

The significance of these interactions is uncertain as they have occurred either in vitro or in excess of clinically relevant concentrations.

### Elimination/excretion

Clearance of cannabinoids is estimated to be 38.8 L/h to 53 L/h (Heuberger et al. 2015), with long terminal half-lives due to their lipophilicity. In regular users, this is extended, with measurable plasma concentrations of THC over 24 h after last administration. Fifteen percent of cannabinoid metabolites undergo enterohepatic recycling, prolonging their action (Ashton 1999).

THC and metabolites are mainly excreted in faeces (65–80%) and urine (20–35%) (Ashton 1999; WHO Expert Committee on Drug Dependence 2018). THC’s elimination half-life is 56 h in occasional and 28 h in chronic users (Ashton 1999) with urinary metabolites measurable 14 days post exposure (Vandrey et al. 2017). Nabilone’s (Cesamet ®; synthetic THC) elimination half-life is shorter than THC, at 2–4 h, yet 16% of a single dose is reportedly measurable at 7 days post administration (Ashton 1999).

CBD metabolites and unchanged drug are mainly excreted in the urine with an elimination half-life of 2–5 days (Lucas et al. 2018; Anderson and Chan 2016; Ujváry and Hanuš 2016).

## Pharmacodynamics (of relevance to the perioperative physician)

### Cardiovascular system

Tachycardia due to CB_1_ agonism in cardiac myocytes has been reported (Kumar et al. 2001; Lucas et al. 2018), but was not noted following intravenous administration of d9THC (Vandrey et al. 2017). Bradycardia, hypotension, an increased cardiac output and myocardial oxygen demand have been described (Dickerson 1980; Bryson and Frost 2011). These effects are potentially exacerbated by sympathomimetic agents although the mechanism of action is unclear (Lucas et al. 2018). Effects may be cannabinoid specific, with CBD (Expert Committee on Drug Dependence 2018) not reported to effect heart rate or blood pressure, and THC possibly having anticholinergic effects through depletion of acetylcholine stores (Dickerson 1980).

### Central nervous system

Effects are well described largely in relation to their abuse as a recreational drug**,** including psychomotor impairment, sedation, dizziness, euphoria, disorientation and confusion. Effects may be enhanced if administered with other CNS depressant drugs, for example opioids or benzodiazepines, and have been observed in a clinical setting (Kumar et al. 2001; Lucas et al. 2018).

The behavioural and long-term psychological effects (including dependence) of cannabis are widely reported (Pertwee 2015; Kumar et al. 2001; Nugent et al. 2017), and not reiterated here. Some evidence suggests the abuse potential of CBMs, likelihood of withdrawal phenomena and mental health morbidity is low (Pertwee 2015; Aragona et al. 2009), but trials are of short duration and do not examine long term effects. Evidence suggests chronic cannabis use impairs learning, memory and attention, and causes complex mental health disorders (Pertwee 2015; Nugent et al. 2017; National Academies of Sciences Engineering and Medicine 2017; Campbell et al. 2018). Further research is needed to determine relevance to CBM use.

### Respiratory system

There is no clear evidence of respiratory system effects when administered by routes other than smoking. This may be due to the absence of cannabinoid receptors in the brainstem (Kumar et al. 2001).

Perioperative practitioners should be alert to the recent warning from the FDA around the use of vaping THC oil (US Food and Drug Administration 2019). This followed on from a multitude of reports of severe pulmonary disease development associated with vaping of THC products (Layden et al. 2019). Any patient presenting in the perioperative period with new onset respiratory disease and a history of vaping THC should therefore be thoroughly evaluated with this kept in mind.

### Immune system

Animal studies suggest that high-dose cannabinoids impair cell-mediated and humoral immunity (Kumar et al. 2001), and low-dose CBD causes immune stimulation (Expert Committee on Drug Dependence 2018). The clinical relevance in humans is unclear.

## Interactions of note for the perioperative physician

### Induction agents/volatiles

Effects of cannabinoids on dosing of volatile and intravenous anaesthetic agents is equivocal, with evidence limited to animal studies, case reports and two limited human studies.

Ether anaesthesia is prolonged in mice and rats by cannabidiol, d8THC and d9THC (Chesher et al. 1974). Halothane anaesthesia is prolonged and dose requirements reduced in dogs after THC administration (Stoelting et al. 1973), with similar effects noted in mice with isoflurane administration (Schuster et al. 2002). Little is known about the interaction between cannabinoids and modern inhalational anaesthetics.

Animal studies have shown cannabidiol, d8THC and d9THC prolong barbiturate anaesthesia in mice and rats (Chesher et al. 1974) and THC administration increases the doses of thiopentone and propofol required for sedation (Brand et al. 2008). A cannabis extract premedication in dogs resulted in quicker onset and longer lasting anaesthesia with propofol (Kumar et al. 2010). One postulated mechanism is the increased Andamide (endocannabinoid) levels in the brain with propofol, with the inhibition of the enzyme fatty acid amide hydrolyses (FAAH), which normally terminates Anandamides activity, thought to be key (Schelling et al. 2006).

There is limited evidence of the effect of cannabinoid exposure on anaesthesia in humans. Case reports suggest increased anaesthetic requirements with non-medicinal cannabis use (Richtig et al. 2015; Symons 2002). A prospective trial found significantly increased propofol dosing for induction and LMA insertion in cannabis users versus controls (Flisberg et al. 2009). Studies utilising bispectral index monitoring (BIS) found no differences between cannabis users and non-users with the bolus dose of propofol required to achieve a BIS of < 60 (Flisberg et al. 2009). Higher BIS values have been noted for patients under steady state volatile anaesthesia who were administered nabiximols (Sativex®) as a premedication versus controls (Ibera et al. 2018).

These results should be interpreted cautiously given the limited number of participants, the applicability of extrapolating animal studies to human practice, use of unknown quantities of non-prescribable CBMs (except one study) and uncertainties about prior cannabis consumption (Flisberg et al. 2009). Additionally in the electroencephalogram (EEG)/depth of anaesthesia studies, it is unclear if the effects are a result of cannabinoids on the EEG or the effect of cannabinoid-anaesthetic interaction.

In summary, there is minimal evidence base as to the effects of the current agents, with animal studies relating to older agents only (ether, halothane, isoflurane). The evidence for intravenous agents is conflicting and of poor quality, but propofol requirements may be higher. There is a current research opportunity for investigation into the interaction with newer agents in humans.

### Opioids

Cannabinoid agonists facilitate endogenous opioid signalling and increase concentrations of endogenous opioids (Scavone et al. 2013; Abrams 2016).

In animal studies (Abrams 2016; Maguire and France 2018), cannabinoids and opioids are synergistic, with the analgesic efficacy of cannabinoids not reduced when opioid antagonists are administered. Human findings are variable; statistically significant reductions in pain scores, and similar opioid pharmacokinetics (with the exception of a reduced C_max_ in the morphine group) pre and post vaporised cannabis use was found in chronic opioid users (morphine/oxycodone) (Abrams et al. 2011). In contrast, a small study found higher pain scores and greater rescue analgesia requirements post operatively in cannabis users, versus non-cannabis users (Jefferson et al. 2013). Chronic cannabinoid and cannabis users undergoing orthopaedic procedures showed higher post-operative pain scores without a significant increase in post-operative opioid consumption (Liu et al. 2018). All these studies have limited numbers of participants, and methodological issues that may confound the results.

In summary, cannabinoids and opioids are synergistic for both wanted and unwanted effects. Chronic cannabis users may have higher pain scores; it is unclear whether this is pathophysiological or a behavioral component of drug use.

### Ketamine

Ketamine induces endogenous cannabinoid release (Pacheco et al. 2019; Ferreira et al. 2018), which may partially explain its role in anti-nociception. The psychomotor side effects of ketamine are enhanced with CBD administration, but no adverse behavioural or cardiovascular effects have been noted (Hallak et al. 2011).

### Gabapentinoids

Gabapentin’s mechanism of action is via α2δ subunits on voltage-dependent calcium channels, with reduction in neural transmission. Similarly, activation of the CB receptor results in inhibition of the voltage dependent calcium channel (Pertwee 2015; Lile et al. 2016). Animal studies have shown the synergistic action of gabapentin and THC when used for the treatment of neuropathic pain, at the expense of increased side effects of THC (Atwal et al. 2017).

Human studies are limited; in multiple sclerosis, the combination of THC and gabapentin improved pain scores in neuropathic pain (Turcotte et al. 2015). High-dose gabapentin for management of cannabis tolerance produces THC like effects, and when gabapentin was used in combination with THC, these effects were seen to be increased, suggesting overlap of pharmacological actions (Lile et al. 2016).

In summary, the gabapentinoids and cannabinoids have overlapping pharmacological actions, with increased therapeutic and side effects when combination dosing is used.

### Dexmedetomidine

There is limited evidence regarding potential interactions between cannabinoids and Dexmedetomidine. Animal studies have shown that a synthetic THC derivative (CP55,940) has additive or synergistic analgesic effects when administered with Dexmedetomidine, depending on the nociceptive stimulus utilised (Tham et al. 2005). The study failed to explain the mechanism of this apparent synergy; however, given the similar intracellular signalling mechanisms (calcium, potassium and cyclic AMP) activated by these medications and the close locality of the target receptors in the periaqueductal grey and substantia gelatinosa, receptor interaction is postulated (Tham et al. 2005).

Given the lack of current evidence around interactions in humans, further research should focus on this area.

## Medical conditions where cannabinoids are recommended

A variety of National and Governmental organisations have provided reviews on the use of CBMs, producing recommendations with a varying hierarchy of evidence (Department of Health and Social Care 2018; Ghebreyesus 2019; Therapeutic Goods Administration 2017; Health Products Regualtory Authority 2017; National Academies of Sciences Engineering and Medicine 2017). Here, we review the commoner indications for CBMs.

### Chronic pain

Information on the use of cannabinoids for chronic pain comes from trials, systematic reviews (SR), meta-analyses (MA) and organisational reports. The outcomes vary, and are limited by factors including study design, moderate to high risk of bias (Hauser et al. 2018a), limited participants (most recent SR/MA (Stockings et al. 2018a) identified 104 studies, 21 with > 100 participants), short duration of exposure to the cannabinoid (median eight weeks (Stockings et al. 2018a)) and varying definitions of “chronic pain”. Many studies within these systematic reviews are notable for high withdrawal rates in the treatment arms (Stockings et al. 2018a; Mucke et al. 2018).

The most recent SR concluded that the number needed to harm (NNTH) for cannabinoid use in chronic pain was 6 (opioids NNTH = 5) (Stockings et al. 2018a) with a number needed to treat (NNT) of 24 (30% reduction in pain). This compares unfavourably with opioids (NNT 4.3), pregabalin (7.7) and tricyclics (NNT 3.6) (Stockings et al. 2018a). When the pain intensity reduction (versus placebo) was pooled, it was equivalent to a 3 mm reduction on a 100 mm visual analogue scale. Taken with a higher risk of an adverse event and trial withdrawal (Stockings et al. 2018a), the authors suggested that whilst there was moderate evidence for pain reduction with cannabinoids compared with placebo (higher quality evidence for MS and neuropathic related pain), it seemed unlikely that cannabinoids are highly effective for chronic non-cancer pain.

Other SR/MAs make varying comments on the strength of the evidence, including weak recommendations (Meng et al. 2017), low strength (Rodríguez-Muñoz et al. 2016), moderate (Therapeutic Goods Administration 2017; Mucke et al. 2018; Whiting et al. 2015) (30% reduction pain relief), moderate to high (Aviram and Samuelly-Leichtag 2017), strong or “conclusive” (Abrams 2018) evidence for the use of cannabinoids in chronic pain. Others suggest a moderate to high risk of bias, concluding the evidence base is insufficient to make well found conclusions about the use of CBMs for cancer and non-cancer pain (Hauser et al. 2018b). Additionally, a large observational cohort study in Australia disputed cannabis use as an adjunct to reduce opiate consumption (Sánchez-Blázquez et al. 2014).

Globally, regulatory bodies have come to different conclusions. The Health Products Regulatory Authority (HPRA) of Ireland does not support CBMs as a treatment in chronic pain (Health Products Regualtory Authority 2017). The European Pain Federations recent position paper recommended CBMs be considered for chronic neuropathic pain, but as a third line agent, and stated there was insufficient evidence for CBMs for non-neuropathic chronic non-cancer pain (Hauser et al. 2018a). This is in direct contrast to the National Academies of Science Engineering and Medicine (NASEM) review on the health effects of cannabis and cannabinoids (National Academies of Sciences Engineering and Medicine 2017) which concluded that there was conclusive or substantial evidence for the use cannabis or cannabinoids for the treatment of pain in adults.

In summary, CBMs have a higher NNT than opioids, pregabalin or TCA, with a clinically insignificant pooled pain reduction of 3 mm on 100 mm VAS, and are thus unlikely to be effective in chronic, non-cancer pain, non-neuropathic pain. Additionally, other problems include study design and high withdrawal rates in intervention arms, with Cannabinoids demonstrating a higher risk of adverse events.

### Nausea and vomiting secondary to chemotherapy

Nabilone (UK) and dronabinol (USA) are used to treat intractable post-chemotherapy nausea and vomiting (Abrams 2018), with the HPRA of Ireland recently permitting its use for this indication (Health Products Regualtory Authority 2017).

Reviews of cannabinoids for this indication have found them to be better than placebo (Whiting et al. 2015; Smith et al. 2015; Layeeque et al. 2006) of similar (Smith et al. 2015; Lewis et al. 1994) or better efficacy than antiemetics (dopamine antagonists) (Whiting et al. 2015), but with patients preferring CBMs (Smith et al. 2015). These reviews do not compare CBMs with steroids or serotonin (5HT_3_) antagonists. One randomised controlled trial utilising ondansetron as a comparator (Meiri et al. 2007) was stopped early due to recruitment difficulties, and had numerous methodological limitations including being underpowered for the authors conclusion that dronabinol was as efficacious as ondansetron.

The quality of evidence for the use of CBMs in preventing chemotherapy induced nausea and vomiting has been described as low (Whiting et al. 2015; Smith et al. 2015), “sometimes effective” (Therapeutic Goods Administration 2017) or conclusive/substantial evidence of benefit (Abrams 2018).

In summary, no completed studies have utilised modern antiemetics as a comparator, but cannabinoids are better than placebo, and display equivalent efficacy with dopamine antagonists. Further research will help determine the appropriate usage of CBM for nausea and vomiting.

### Multiple sclerosis

Nabiximols (Sativex®) is licensed for multiple sclerosis (MS)-induced spasticity (Department of Health and Social Care 2018), which affects 17% of MS sufferers, with a similar proportion using cannabis for symptom control (Rice and Cameron 2017).

Previous MA/SR have produced various conclusions on the strength of the evidence of CBMs in MS-induced spasticity, ranging from low quality to conclusive evidence (Therapeutic Goods Administration 2017; Health Products Regualtory Authority 2017; Rice and Cameron 2017; Koppel et al. 2014; Whiting et al. 2015; Abrams 2018). A recent systematic review of reviews (Nielsen et al. 2018) for the use of cannabinoids in MS concluded that whilst the quality of the evidence from included studies was very low to low, five of the eleven reviews concluded that there was sufficient evidence for reduction in spasticity and/or pain in MS. However, the authors stated that despite the positive findings, the effect was small (Nielsen et al. 2018).

In summary, CBMs have a small positive effect on muscle spasticity, but the evidence quality is low.

### Epilepsy

The United States Food and Drug Administration (FDA) (US Food and Drug Administration 2018) has recently approved cannabidiol oral solution (Epidiolex®) for the treatment of two forms of rare epilepsy in children aged over 2 years of age; Lennox-Gastaut syndrome and Dravet syndrome (Thiele et al. 2018; Devinsky et al. 2018; Devinsky et al. 2017). Most evidence is on the use of CBD, with the overall quality of the evidence in adults being limited (Koppel et al. 2014; Abrams 2018; Gloss and Vickrey 2014; Stockings et al. 2018b). Meta-analysis results pool effects in adults and children, with conclusions being influenced by the aforementioned paediatric studies (Sánchez-Blázquez et al. 2014; Devinsky et al. 2018; Devinsky et al. 2017; Gloss and Vickrey 2014; Stockings et al. 2018b). Outside of the USA, CBM use for epilepsy is not recommended in the UK (Department of Health and Social Care 2018), Ireland (Health Products Regualtory Authority 2017) and only once conventional treatments have failed in Australia (Therapeutic Goods Administration 2017).

In summary, the evidence base supports the use of CBD in children with certain neurological conditions, but not in adults.

## Other

The NASEM review has considered CBMs for the treatment of other conditions, as detailed in Table 3 (Abrams 2018).

**Table 3:** Potential indications for CBM and evidence base

Evidence of benefit	Condition
Moderate	-Improving short-term sleep in sleep disturbance associated with OSA, Chronic pain, MS
Limited	-Improving symptoms of post-traumatic stress disorder, anxiety -Improving appetite and decreasing weight loss associated with HIV/AIDS -Tourette syndrome
None	-Treatment of cancer -Irritable bowel syndrome symptoms -Neurodegenerative conditions -Addiction

## Potential future uses in perioperative medicine

### Nausea and vomiting prophylaxis and treatment

There is a paucity of evidence on the effects on post-operative nausea and vomiting (PONV). Nabilone (Cesamet®) (Lewis et al. 1994; Levin et al. 2017) and intravenous THC (Kleine-Brueggeney et al. 2015) have been shown to be ineffective for PONV. Nabilone premedication compared with placebo (Levin et al. 2017) or metoclopramide (Lewis et al. 1994) had no effect on PONV. Intravenous THC similarly showed a lack of effect, with early trial cessation due to an intolerable side effect profile (Kleine-Brueggeney et al. 2015).

Combination therapy (dronabinol and prochlorperazine) compared with routine care showed a reduction in the incidence of PONV, yet the retrospective nature and multiple confounders means the evidence has to be carefully interpreted (Layeeque et al. 2006).

### Perioperative pain management

A systematic review looking at the efficacy of cannabinoids for acute pain management suggested no role for cannabinoids (Stevens and Higgins 2017).

For perioperative pain management, a small number of RCTs have been conducted with two studies suggesting benefit (Jain et al. 1981; Holdcroft et al. 2006). The first (Jain et al. 1981) showed significantly improved post-operative pain scores compared with placebo, but at the expense of increased side effects including drowsiness and dysphoria. The second (Holdcroft et al. 2006), a dose escalation study, with an oral capsule mixture of THC/cannabidiol (and other plant-based cannabinoid extracts) reported a similar NNT as other rescue analgesics, but with significantly increased side effects including sedation and nausea. The applicability of these results is limited by methodological issues and the small number of participants.

Six other studies (Ostenfeld et al. 2011; Beaulieu 2006; Buggy et al. 2003; Seeling et al. 2006; Kalliomäki et al. 2013; Guillaud et al. 1983) investigating the perioperative use of CBMs for analgesia showed no improvement in pain scores; one (Beaulieu 2006) showing significantly higher pain scores.

If CBMs are to be introduced into the clinical pharmacopoeia for perioperative analgesia, the potential for synergy with concurrently administered opioids (especially slow release formulations) in the perioperative period should be considered. One of the main concerns, and as recently highlighted by both the Anaesthesia Patient Safety Foundation (APSF) and ANZCAs faculty of pain medicine (Anaesthesia Patient Safety Foundation 2018; Australia and New Zealand College of Anaesthetists Faculty of Pain Medicine 2018) is the potential for opioid induced ventilatory impairment (OIVI) (Australia and New Zealand College of Anaesthetists Faculty of Pain Medicine 2018). We would recommend sedation soring be undertaken in these patients, as well as standardised order sets as recently recommended by the APSF (Anaesthesia Patient Safety Foundation 2018) and ANZCA (Australia and New Zealand College of Anaesthetists Faculty of Pain Medicine 2018).

In summary, cannabinoids may improve pain relief as part of multi-modal approach. There is an increased risk of adverse side effects including increased sedation and subsequent ventilatory impairment.

### The future of CBMS

Further clarification on the role of non-CBD CBMs is expected later this year with the forthcoming UNODC vote on rescheduling as recommended by the WHO, increasing the focus on this group of medicines. With time, this may help to improve the evidence base, define clinical indications including potential therapeutic applications in perioperative medicine and provide outcome data from longer term use, which is currently lacking (Health Products Regualtory Authority 2017; Fitzcharles and Eisenberg 2018).

This latter point is arguably the most important, and whilst cannabis use per se has been associated with some cancers (prostate, glioma, cervical) and psychiatric morbidity, the quality of the evidence is limited, and it is uncertain if long-term effects of CBMs can be extrapolated from long term cannabis use (Nugent et al. 2017; National Academies of Sciences Engineering and Medicine 2017; Campbell et al. 2018).

Therefore, further research is required, and whilst one of the longest follow up studies of cannabis use in a medical setting (Ware et al. 2015) suggested no difference in serious adverse events between controls and cannabis users, the short duration of this and other studies involving CBMs limits conclusions on long-term safety (Hauser et al. 2018c). Long-term data on CBMs is now being collected through patient registries (national and pharmaceutical led) and observational studies providing reporting of adverse effects (Krcevski-Skvarc et al. 2018). Achieving greater clarity on the benefits and harms of CBMs may be affected by the legalisation of cannabis for recreational use in some territories (United Nations Office on Drugs and Crime 2018).

## Conclusions

There are marked discrepancies in the literature regarding grading of the evidence base and the strength and quality of the resultant recommendations.

It is clear that with the increasing trend for legalisation of this class of medicines, and the large number of patients we as a specialty are involved with, the perioperative team need to have a broader understanding of the pharmacology interactions, and potential uses this group of drugs has.

As the evidence base increases, CBMs could become part of the perioperative teams’ armamentarium to help provide an opiate sparing multimodal analgesia regime as well as having a role in the management of common post-operative complications such as nausea and vomiting.

## Data Availability

Not applicable.

## Abbreviations

BIS: Bispectral Index
CB: Cannabinoid
CBD: Cannabidiol
CBN: Cannabinol
CBM: Cannabis-based medicines
CYP: Cytochrome P450
D8THC: Delta-8-tetrahydrocannabinol
D9THC: Elta-9-tetrahydrocannabinol
EEG: Electroencephalogram
GABA: Gamma amino butyric acid
HPRA: Health Products Regulatory Authority
MA: Meta-analysis
MS: Multiple sclerosis
NASEM: National Academies of Science Engineering and Medicine
NNT: Number needed to treat
NNTH: Number needed to harm
PONV: Post-operative nausea and vomiting
RCT: Randomised controlled trial
SR: Systematic review
THC: Tetrahydrocannabinol
UK: United Kingdom
USA: United States of America
WHO: World Health Organisation
